# Science and Facebook: The same popularity law!

**DOI:** 10.1371/journal.pone.0179656

**Published:** 2017-07-05

**Authors:** Zoltán Néda, Levente Varga, Tamás S. Biró

**Affiliations:** 1 Babeș-Bolyai University, Department of Physics, Cluj-Napoca, Romania; 2 HIRG, HAS Wigner Research Centre for Physics, Budapest, Hungary; Universidad de las Palmas de Gran Canaria, SPAIN

## Abstract

The distribution of scientific citations for publications selected with different rules (author, topic, institution, country, journal, etc…) collapse on a single curve if one plots the citations relative to their mean value. We find that the distribution of “shares” for the Facebook posts rescale in the same manner to the very same curve with scientific citations. This finding suggests that citations are subjected to the same growth mechanism with Facebook popularity measures, being influenced by a statistically similar social environment and selection mechanism. In a simple master-equation approach the exponential growth of the number of publications and a preferential selection mechanism leads to a Tsallis-Pareto distribution offering an excellent description for the observed statistics. Based on our model and on the data derived from PubMed we predict that according to the present trend the average citations per scientific publications exponentially relaxes to about 4.

## Introduction

The number of citations for a publication is basically a social popularity measure for it, while it is considered to reflect the quality and impact of the research.

Citations are thus in our focus when evaluating researchers, groups and institutes [1–3]. The statistics and dynamics of citations are studied in several works [4–9] and lately we assisted to many serious debates on their use for quantifying objectively the quality and impact of a given research [1–3, 10–13]. In view of this, further scientific arguments or novel information regarding the citation statistics and its similarity to other social selection mechanisms is of enhanced importance.

**Fig 1:**
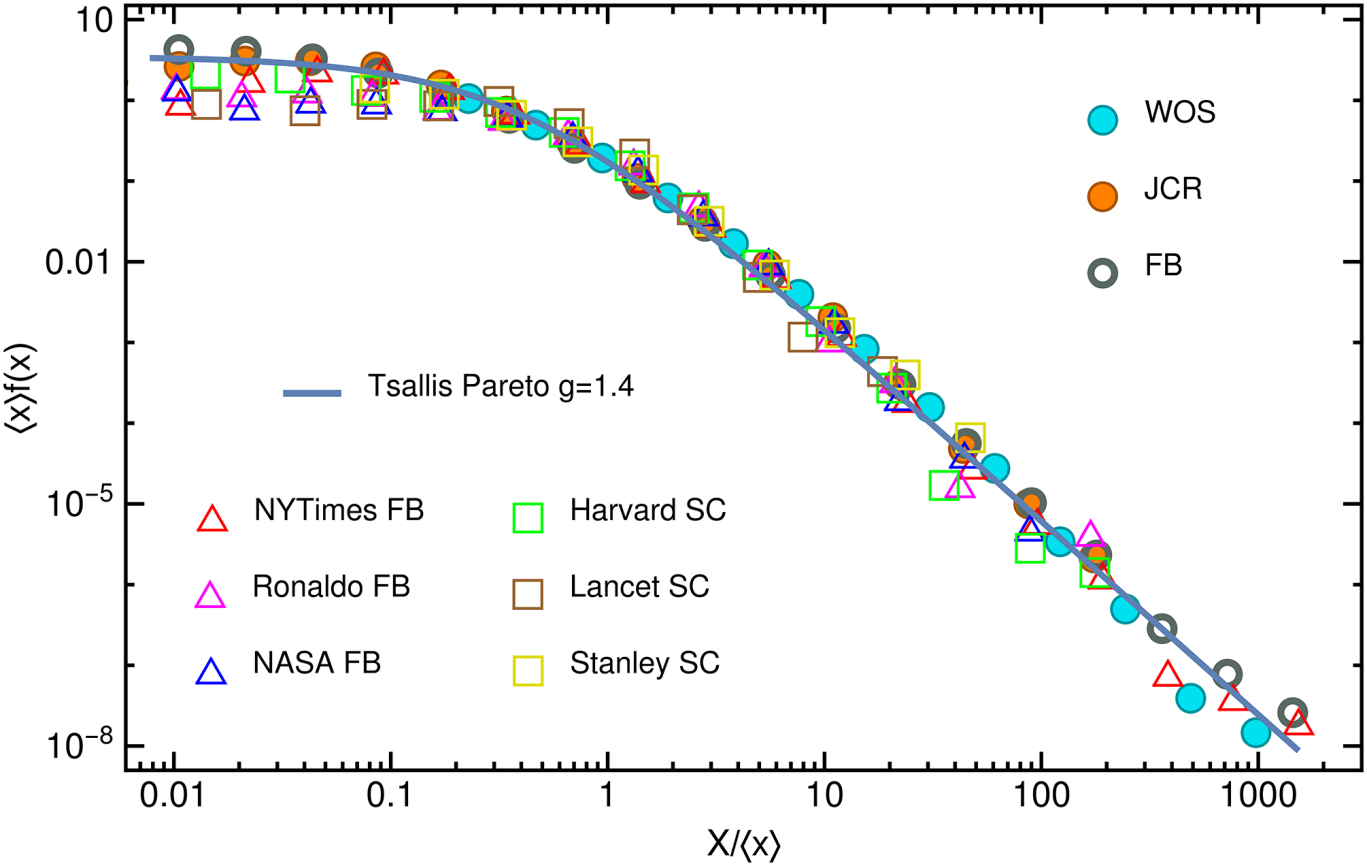
Rescaled distribution of the citation (share) numbers. *f*(*x*) is the probability density (PDF) for one paper (post) to have *x* citations/shares. We present the 〈*x*〉 ⋅ *f*(*x*) value as a function of *x*/〈*x*〉 (〈*x*〉 the mean value, or first moment of the PDF). For high citation number a clear power-law trend is visible. Different symbols are for different datasets as illustrated in the legend. The considered datasets are described in the Methods section. For high *x*/〈*x*〉 a clear power-law trend is visible. The entire curve can be well-fitted with a TP distribution Eq (1) with *g* ≈ 1.4 and 〈*x*〉 = 1.

It has been reported [4–6] that citations for scientific papers, selected according to an arbitrary collection rule, like author, topic, publication year, institution, journal, etc…, rescale on a common curve if considering their value relative to the average. More specifically, if one computes for the selected set the probability density *f*(*x*) for one paper to have *x* citations, and represent graphically the 〈*x*〉 ⋅ *f*(*x*) value as a function of *x*/〈*x*〉, the data obtained for different sets will collapse on the same curve (see the figures in [4–6] and Fig 1). We denoted here with $\langle x\rangle =\int_{0}^{\infty }x\;f\left(x\right)\;dx$, the mean value of *x*, or the first moment of the probability distribution function (PDF). For high citation numbers a clear power-law trend is visible, especially if one considers datasets where the *x*/〈*x*〉 > 10 domain is visible. There is, however, a very active debate on fitting this rescaled curve [4–6, 9, 14–19]. Researchers have suggested lognormal, negative binomial, Wakeby and power-law tailed distributions to fit the entire curve. Recent results [16, 18, 19] favour a Tsallis-Pareto (TP) [20, 21] type hooked distribution, albeit the lognormal distribution is still in use [6]. The obvious scale-free nature of the tail and accordingly the observed invariance relative to mixing or selecting just a part of the ensemble is however a major argument in favour of the TP distribution. It worth noting here that recently it has been shown [22] that the frequency distribution of scientific memes follows also a universal distribution with a power-law like tail. In such aspect the TP distribution could be a good candidate for fitting also the results presented in [22].

Biology, physics and socio-economic phenomena offer many intriguing examples of scale-free distributions in complex systems [23–25]. The celebrated Zipf law [26], or many other power-law tailed distributions are widely known and well-studied [27]. The pure power-law, however, is not a distribution in the strict mathematical sense since it cannot be normalized for the whole interval between zero and infinity. Quite frequently we do not even have a large enough scaling interval to prove or disprove the presence of pure power-law distributions [28]. On the other hand the Tsallis-Pareto distribution [20, 21]

$$\begin{array}{c} f\left(x\right)=\frac{g}{\left(g-1)\langle x\right\rangle }{\left(1+\frac{x}{\left(g-1)\langle x\right\rangle }\right)}^{-1-g} \end{array}$$ (1)

is a proper probability density function (PDF) with a power-law like tail. It has been found that many heavy-tailed distributions are well fitted by using the above PDF [24]. Although this is not strictly a scale-free distribution, one can numerically check that for *g* > 1 exponents and for large enough *x*/〈*x*〉 the scale free properties and invariance under mixing or splitting of the dataset are well satisfied.

## Results and discussions

A simple exercise on citation data collected from more than 600 000 ISI Web of Science (WOS) publications (mapping a part of the WOS citation network by using an Internet robot, please see the Methods section), draws the shape of the universal form for the studied distribution. If one makes a simple data processing exercise from the total number of citations received in ten years for all ISI indexed journals (InCites, Journal Citation Reports [29]), the data (JCR) scale on the very same curve. If we select now data for the publications authored by one researcher, for the publications published in a given journal in one given year or by authors associated to a given institute, the data rescale again. For *x*/〈*x*〉 ≥ 0.1 the collapsed data can be nicely fitted with a one-parameter TP type PDF, using *g* ≈ 1.4 and 〈*x*〉 = 1. (see Fig 1). As already emphasised, this type of fit has the advantage that the scale-free property for *x*/〈*x*〉 ≥ 0.1 is evident and also explains the invariance of the distribution when combining several data sets.

A similar study can be performed on different Facebook pages for their posts (for details please consult again the Methods section). Instead of citations the popularity proxy for a post is the number of “shares” it receives. “Share” is a stronger selection rule than the simple “like”, and it’s role is similar to citations in Science. Interestingly the PDF for “shares” collected from 16 different Facebook users (in total more than 150 000 posts) scale on the very same curve with the Scientific Citations (Fig 1). The universal TP type distribution with *g* ≈ 1.4 suggests a common growth mechanism for the Facebook Shares and Scientific Citations. Reducing now the Facebook data on users, the rescaled PDF behaves in a similar manner (for details on the used data see again the Methods section). Due to the larger scatter for the data points resulting from the reduced data size (both for scientific citations and Facebook shares) we cannot conclude however the same Pareto exponent, just note the similar trend. The invariance of the distributions relative to the splitting of the data is in agreement with the scale-free properties of this distribution.

The appropriateness of the TP type fit can be proved by computing the generally used *R*^2^ *coefficient of determination*. We have computed this for the datasets that contained a fair number of elements (e.g. the statistics is reliable): WOS, JCR and FB. The *R*^2^ > 0.9 values presented in Table 1 indicates that the TP type fit is justified. For the smaller datasets such statistics is not relevant, and one can just note that the data points are following the trend of the TP fit with *g* = 1.4.

**Table 1:** Coefficient of determination for the TP fit in Fig 1.

	WOS	JCR	FB
*n*	629 575	12 026	160 889
**R**^**2**^	0.982	0.967	0.940

Goodness of fit for the large datasets (WOS, JCR and FB) in Fig 1. We denote by *n* the size of the dataset and by *R*^2^ the coefficient of determination. The fit is given by the TP distribution, Eq (1), with *g* = 1.4 and 〈*x*〉 = 1. For more details on the experimental data please consult the Methods section.

Many models have been already considered for explaining the dynamics of citations [30–32] and the observed universality in the rescaled PDF [33, 34]. A simple explanation for this intriguing universality can be given by considering a coarse-grained master equation for the growth process and assuming an exponential growth of publications (post) number as a function of time together with a linear preferential growth rate in the flow (for details please see the technical subsection: The Master equation approach).

The approach considered here is the simplest mean-field type approximation where only the stochastic nature of the growth process is taken into account and the specificity of the posts quality are coarse-grained. The exponential growth of the number of publications which are the carriers of the citations is known (see for example [35, 36]). In a recent statement form Mark Zuckerberg we also learn that the information sharing activity on Facebook is also growing exponentially (see for example [37]).

On the other hand the linear preferential growth rate hypothesis or the commonly known Matthew effect (“For to all those who have, more will be given”) has been highlighted in various social systems [38–40]. The presence of the Matthew effect in citation and science was also discussed in many previous publications [41, 42]. In such manner the two main assumptions of our simple model are all reasonable, and can be applied both to Facebook posts and scientific articles. The Markov-like process constructed on these bases can be analytically solved also in the continuous limit where it leads to a TP Eq (1) probability distribution (see the technical subsection: The Master equation approach). From the model we learn that the parameter *g* in the TP distribution, governing the power-law tail, is just the ratio of the exponential growth rate *γ* to the proportionality constant *σ* for the linear preferential growth: *g* = *γ*/*σ*. The fact that the obtained *g* value is independent from the way we construct the studied ensemble and it is the same for Facebook posts and Scientific Publications is intriguing. It can be understood by taking into account that both phenomena are taking place on a social network with similar topological properties, where the released information amount is increasing exponentially and the selection rules for its transmission are adapted to the increase rate.

### The Master equation approach- technical details

**Fig 2:**
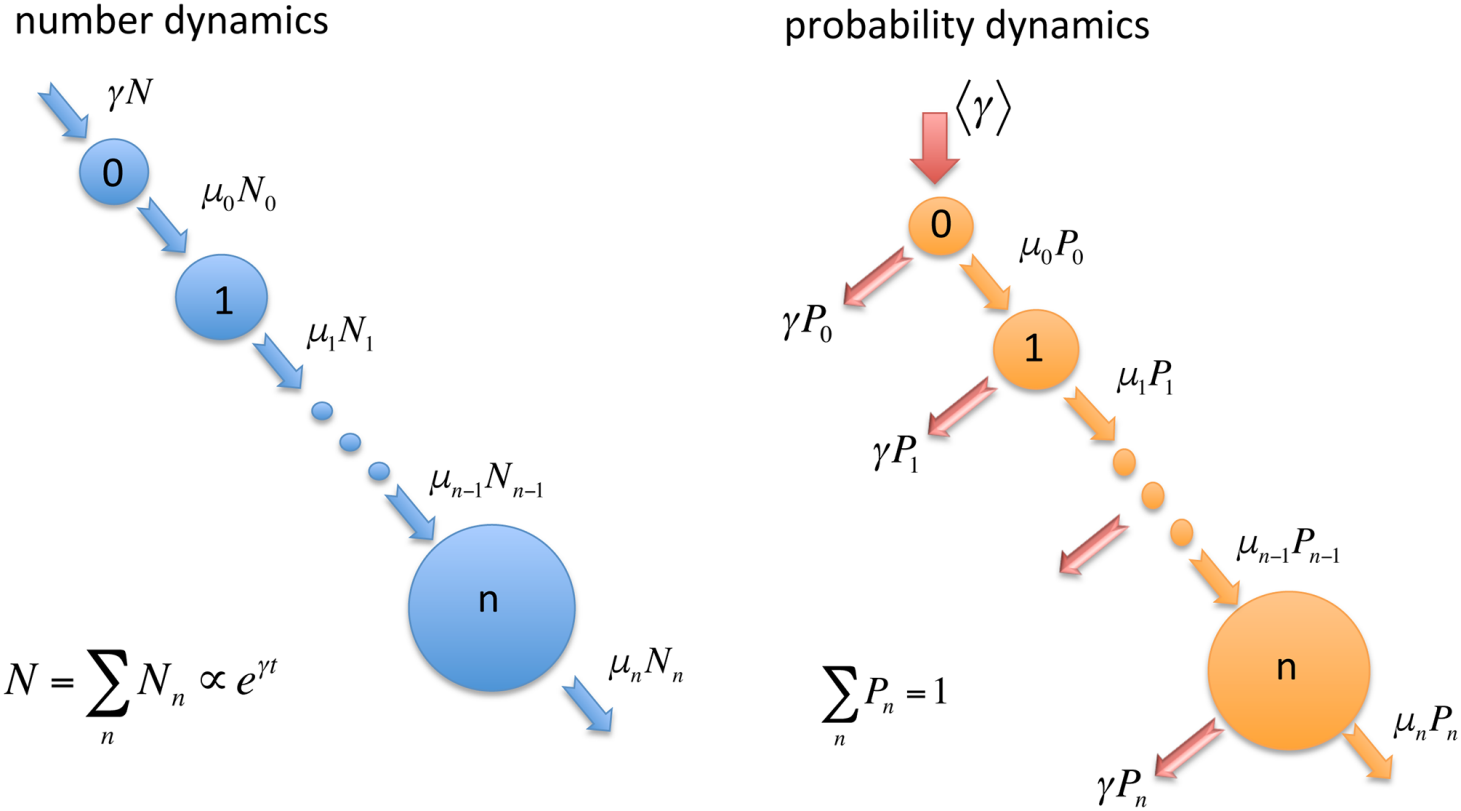
Schematic representation of the coarse-grained random growth model. The panel on the left side indicates the growth process in the number of elements with *n* quanta: *N*_*n*_. Due to the fact that the total number of elements is exponentially increasing, the probability *P*_*n*_ that an element will have *n* quanta, experiences the dynamics sketched on the right panel of the figure.

We consider a classical master equation approach for the growth phenomenon. This approach is the simplest possible mean-field like description where the properties of different elements (posts, publications) are coarse-grained and only the stochastic character of the process is kept. In this framework, the stochastic growth process is quantified by a mean growth rate *μ*_*n*_ describing the transition rate from state with *n* quanta (citations, shares, likes…) to a state with *n* + 1 quanta. Since there is no reverse process inside the chain, just a continuous growth a detailed balance condition cannot be fulfilled. We illustrate this process in the left panel of Fig 2, where *N*_*n*_(*t*) denotes the number of elements having *n* quanta at time moment *t*. A master equation for this process writes as:

$$\begin{array}{c} \frac{dN_{n}\left(t\right)}{dt}=\mu_{n-1}N_{n-1}\left(t\right)-\mu_{n}N_{n}\left(t\right) \end{array}$$ (2)

In order to achieve a non trivial steady-state distribution, parallel with this continuous growth a continuous dilution should be present in the system. This can be achieved by assuming that the number of elements are continuously increasing in time. This means that

$$\begin{array}{c} N\left(t\right)=\sum_{n}N_{n}\left(t\right) \end{array}$$ (3)

is increasing in time. Considering now the probability *P*_*n*_(*t*) that an element has *n* quanta at time moment *t*

$$\begin{array}{c} P_{n}\left(t\right)=\frac{N_{n}\left(t\right)}{N}\;, \end{array}$$ (4)

we rewrite the master equation using instead of *N*_*n*_(*t*) the *P*_*n*_(*t*) distribution:

$$\begin{array}{c} \frac{d}{dt}\left(NP_{n}\right)=N\frac{dP_{n}}{dt}+P_{n}\frac{dN_{n}}{dt}=\mu_{n-1}NP_{n-1}-\mu_{n}NP_{n} \end{array}$$ (5)

The number of elements in the considered systems is exponentially increasing. Assuming thus an exponential growth in *N*(*t*) with a rate *γ*, characteristic for each ensemble in part (scientific papers, Facebook posts, etc…):

$$\begin{array}{c} N\left(t\right)=N\left(0\right)e^{\gamma t}\to \frac{dN(t)}{dt}=\gamma N\left(t\right), \end{array}$$ (6)

From Eq (5) we arrive now to a master equation in *P*_*n*_(*t*):

$$\begin{array}{c} \frac{dP_{n}}{dt}=\mu_{n-1}P_{n-1}-\left(\mu_{n}+\gamma \right)P_{n} \end{array}$$ (7)

The flow diagram for this process is illustrated in the right panel of Fig 2. The corresponding equation for the *n* = 0 term can be obtained from the normalization condition ∑_*n*_ *P*_*n*_(*t*) = 1:

$$\begin{array}{c} \frac{dP_{0}}{dt}=\gamma -\left(\mu_{0}+\gamma \right)P_{0} \end{array}$$ (8)

We consider now the continuous limit of Eq (7) (see for example [4]), where the discrete states *n* are replaced by continuous *x* states:

$$\begin{array}{c} \frac{\partial P(x,t)}{\partial t}=-\frac{\partial }{\partial x}\left(\mu \left(x\right)P\left(x,t\right)\right)-\gamma P\left(x,t\right)+\gamma \delta \left(x\right) \end{array}$$ (9)

This equation describes a flow with a general velocity field *μ*(*x*), a loss rate *γ* and a feeding at *x* = 0. (We denoted by *δ*(*x*) the Dirac functional). The *P*_*s*_(*x*) stationary probability density can be derived from the condition: $\frac{\partial P_{s}\left(x,t\right)}{\partial t}=0$, and according to Eq (9) it satisfies

$$\begin{array}{c} \frac{d}{dx}\left(\mu \left(x\right)P_{s}\left(x\right)\right)=-\gamma P_{s}\left(x\right)\;. \end{array}$$ (10)

The solution of this equation writes as

$$\begin{array}{c} P_{s}\left(x\right)=\frac{K}{\mu (x)}e^{-\gamma \int \frac{1}{\mu (x)}dx} \end{array}$$ (11)

In order to write up the solution one has to specify a kernel for the *μ*(*x*) growth rate. From several social-economic phenomena we learn that the growth is usually governed by a preferential selection, in the simplest case by a linear preferential growth rate (the well-known “rich gets richer” phenomenon or the Matthew effect [38, 39]). According to this

$$\begin{array}{c} \mu (x)=\sigma (x+b)\;, \end{array}$$ (12)

where the *σ* and *b* values are characteristic to the considered ensemble (scientist, Facebook users). Accepting this kernel, Eq (11) leads to the Tsallis-Pareto distribution [20, 21]:

$$\begin{array}{c} P_{s}\left(x\right)=\frac{\gamma }{b\sigma }{\left(1+\frac{x}{b}\right)}^{-1-\gamma /\sigma } \end{array}$$ (13)

Denoting *g* = *γ*/*σ* and using *b* = 〈*x*〉(*g* − 1), where 〈*x*〉 is the first moment of the distribution, we get:

$$\begin{array}{c} P_{s}\left(x\right)=\frac{g}{\left(g-1)\langle x\right\rangle }{\left(1+\frac{x}{\left(g-1)\langle x\right\rangle }\right)}^{-1-g} \end{array}$$ (14)

This is the scaling Tsallis-Pareto distribution, which for *g* = 1.4 and 〈*x*〉 = 1 offers a good fit for the collapsed data on Fig 1. The prediction of our simple model is in agreement with the more technical approach considered in [32].

From this simple mean-field type model we learn that the popularity measures both for scientific publications and Facebook are the results of an exponential growth and a preferential retransmission of the received information. The collapse for the Facebook popularity measures and scientific citations indicate that for their coarse-grained dynamics the ratio *g* = *γ*/*σ* should be similar. Seemingly this ratio is also independent on the precise manner in how we construct the ensembles (institutes, journals, individuals, etc…). This is an exciting finding which inspires further studies.

### Trend for the average number of citations per paper

From the promising fit indicated in Fig 1, using *g* ≈ 1.4, we gain confidence in the statistical prediction capability of our simple mean-field type approximation. We elaborate thus further on our model and make some statistical predictions on the expected evolution of the average number of citations (shares) per publication (post). The total number of citations at time *t* can be written as: *C*(*t*) = ∑_*n*_ *nN*_*n*_(*t*). According to our hypothesis the increase in the total number of citations in unit time is given as:

$$\begin{array}{c} \frac{dC(t)}{dt}=\sum_{n}\frac{dN_{n}^{+}}{dt}=\sum_{n}\sigma \left(n+b\right)N_{n}=\sigma C\left(t\right)+\sigma bN\left(t\right) \end{array}$$ (15)

Combining this with the exponential growth of *N*(*t*): $\frac{dN(t)}{dt}=\gamma N\left(t\right)$ leads to a simple differential equation for the *m*(*t*) = *C*(*t*)/*N*(*t*) average number of citations per work: $\frac{dm(t)}{dt}=\left(\sigma -\gamma \right)m\left(t\right)+\sigma b$. The solution is an exponential relaxation

$$\begin{array}{c} m\left(t\right)=\frac{K}{\gamma -\sigma }e^{-(\gamma -\sigma )t}+\frac{b}{g-1}\;, \end{array}$$ (16)

where *K* is an integration constant. Due to the fact that *g* = *γ*/*σ* ≈ 1.4 we get *γ* > *σ* and therefore *c*(*t*) has an exponentially relaxing trend. From the previous section we learned that *b*/(*g* − 1) = 〈*m*〉_*ech*_, the equilibrium value for the average citation per paper in the considered ensemble.

We can now determine the time-evolution of the total citations number per year. Let us assume now that we measure the time in years, and introduce the yearly published article number $n\left(t\right)=\frac{dN(t)}{dt}=n_{0}e^{-\gamma (t-t_{0})}$, and the new citations that appear in one year: $c\left(t\right)=\frac{dC(t)}{dt}$. If we assume that at time *t*_0_ we have *c*(*t*_0_) = *c*_0_ and *n*(*t*_0_) = *n*_0_ we get that

$$\begin{array}{c} K=\left(\frac{c_{0}}{n_{0}}\left(g-1\right)-b\right)\frac{\gamma }{g}\;, \end{array}$$ (17)

and

$$\begin{array}{c} c\left(t\right)=c_{0}e^{\sigma (t-t_{0})}+\frac{bn_{0}}{\left(g-1\right)}\left(e^{\gamma (t-t_{0})}-e^{\sigma (t-t_{0})}\right)\;. \end{array}$$ (18)

**Fig 3:**
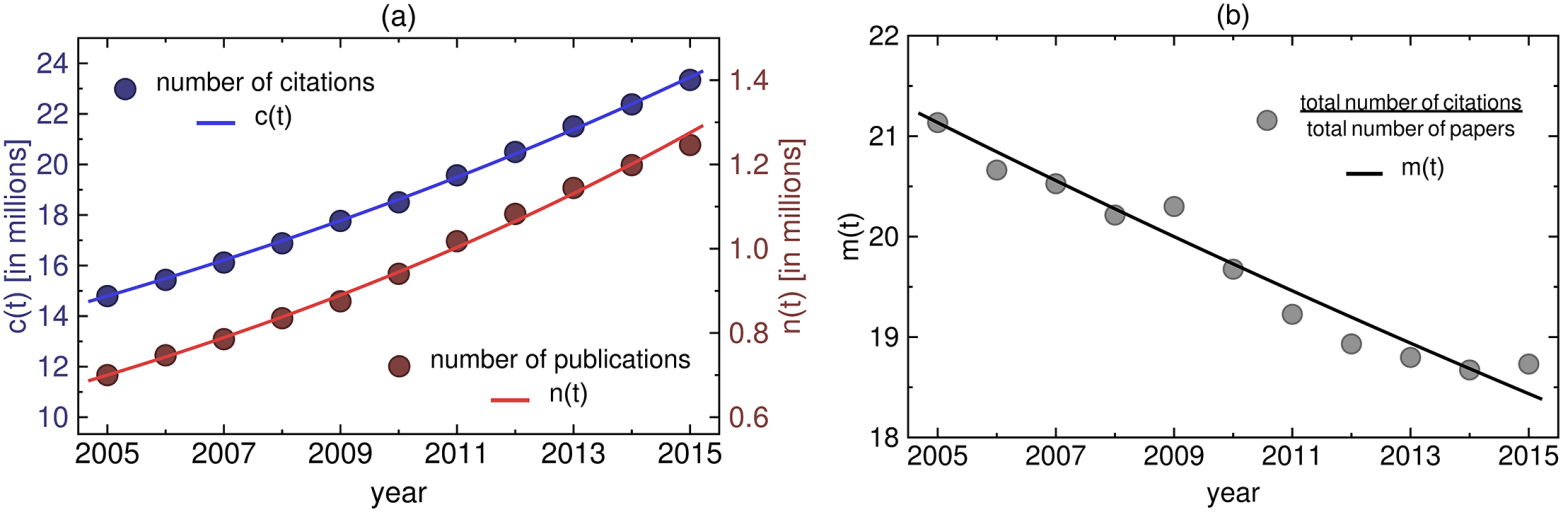
Results for the MEDLINE/PubMed database. **Fig 3(a)** illustrates the time evolution of the yearly indexed papers, *n*(*t*), and the total number of citations, *c*(*t*), introduced by them for each year in the 2005-2015 time interval. The trend *n*(*t*) can be nicely fitted (red curve) with an exponential curve with *γ* = 0.06 using *t*_0_ = 2005 and *n*_0_ = 699 915. For *t*_0_ = 2005, *n*_0_ = 699 915, *c*_0_ = 14 792 864, *g* = 1.4 and *γ* = 0.06 (*σ* = *γ*/*g* ≈ 0.043) the trend given by Eq (18) can be approximated with *b* ≈ 1.6. **Fig 3(b)** illustrates the time evolution for the yearly incoming citations divided by the total number of new papers, *m*(*t*). Using the parameters from *n*(*t*) and *c*(*t*), the *m*(*t*) trend given by Eq (16) is plotted by the black curve.

For the case of scientific articles indexed in MEDLINE/PubMed (see the Methods section) we can determine the *γ* ≈ 0.06 value (Fig 3(a)), which leads to *σ* ≈ 0.043. A simple fitting exercise on the *c*(*t*) curve using the data for PubMed, leads us to *b* ≈ 1.6 (Fig 3(a)). According to these results we predict that the average number of citations per article (for the case of PubMed indexed articles) will relax to *b*/(*g* − 1) ≈ 4 (Fig 3(b)).

## Conclusion

Our **conclusions** are pretty clear: Science and Facebook show the same popularity pattern which can be simply understood by a coarse-grained master equation approach where we admit the exponentially increasing amount of information together with a “rich gets richer” preferential information filtering mechanism. Our model predicts that the average number of citations per publication (or shares per Facebook posts) exponentially relaxes to a constant value. This suggests that our society acts in responsible and selective manner in retransmitting the informations. For scientific articles we predict that their average number of citations converges to a value of approximately 4.

## Methods

The data plotted in Fig 1 was collected as follows:

For the WOS dataset (Scientific Citations from ISI Web of Science) we used an Internet robot that started form a given article and reached all the papers that were cited by this. We extracted only the article’s identification code and have done this for a depth of four levels recording the total number of citation for all ISI indexed articles that were accessed with this procedure. In total more than 600 000 articles were reached. The use of such robots for accessing an incomplete part (only accession numbers) of the database is not prohibited by the terms of use for the Web of Science [43].

For the JCR dataset we have downloaded the table from InCites, Journal Citation Reports [29], and recorded “total number of citations” for each of the (more than 12 000) indexed journals. For the reduced datasets we followed the methodology described in [6] selecting by random some Institutes, Journals and researchers. We extracted from ISI Web of Science the citations up to the present date for articles published in 1990 with authors from Harvard University. In the same manner for journals we selected papers published in The Lancet (Elsevier) in 1990 and recorded their citations up to the present date. Since our results were in agreement with the one published in [6], we concluded that the results for other Institutes and Journals rescale on the very same curve as it is illustrated in [6]. To complete the study on citation distribution with an even more challenging dataset we have selected a single author from physics (Prof. H. E. Stanley from the Boston University, USA) with an impressive number of publications (965 ISI papers) and ISI citations (62 996) and constructed the citation distribution for all his papers up to the present date independently of the publication year.

In collecting the statistics for MEDLINE/PubMEd articles we have used the trend for the total number of publications from [44], and the yearly total number of citations statistics from [45].

For Facebook we used only public pages and informations that are publicly visible. In order to do this in an automatic manner we registered as a developer, and used a publicly available page scraper [46]. Since all collected data are already public on Facebook no privacy issues were violated, for more information on this page scraper please see the relevant information available at: [46]. We have selected 16 Facebook pages that have a relatively high number of shares for their posts in comparison to other users in the same field of activity. For news we selected: New York Times, CNN, BBC news; for science we selected: NASA, and National Geography; for sport celebrity we selected Cristiano Ronaldo; for festivals we have chosen Burning Man, for nightclubs: Sugar Factory; for administration: USA gov., European Council, European Parliament; for movies and TV celebrities: IMDB and for politics: Democratic Party of USA and the Republican Party of USA. From their metadata we have extracted the number of “shares” for all posts independently of their publication date. We have also combined all “share” numbers for the posts of all 16 users and considered as the combined FB database.

All data used to plot the figures are available for download [47].

Probability distribution functions were constructed using a logarithmic binning method, considering bins of sizes 2^*n*^. In order not to overload Fig 1 we have plotted the results only for some selected datasets (see the legend). The other collected data, follows the same general trend. All the rescaled data can be nicely fitted with a TP distribution with *g* = 1.4 and 〈*x*〉 = 1.

## Privacy statement for the collected data

All data collected on Facebook are publicly available, and no other personal data was collected. No privacy issues were violated.
